# A Low-FODMAP Diet in the Management of Children With Functional Abdominal Pain Disorders: A Protocol of a Systematic Review

**DOI:** 10.1097/PG9.0000000000000065

**Published:** 2021-03-30

**Authors:** Agata Stróżyk, Andrea Horvath, Hania Szajewska

**Affiliations:** Department of Paediatrics, The Medical University of Warsaw, Warsaw, Poland.

**Keywords:** irritable bowel syndrome, diet, abdominal pain, oligosaccharides, fructose

## Abstract

The available interventions for the management of children with functional abdominal pain disorders (FAPD) are limited. A diet low in fermentable oligosaccharides, disaccharides monosaccharides, and polyols (FODMAPs) is widely used in adults and children with FAPD, despite limited available evidence. We aim to systematically review evidence on the efficacy and safety of using a low-FODMAP diet for the management of children with FAPD.

**Methods:**

The Cochrane Library, EMBASE, and MEDLINE databases will be searched for randomized controlled trials (RCTs) that compare the use a low-FODMAP diet (preferably a 3-step low-FODMAP diet but also only a strict low-FODMAP diet or restriction of individual FODMAPs) with any comparator (i.e., standardized [i.e., average national] or other diet or no intervention) in children with FAPD (regardless of the definition). Each FAPD and each low-FODMAP diet or individual FODMAP restriction will be assessed separately. The Cochrane Collaboration’s tool for assessing the risk of bias will be used. The primary outcome will be the abdominal pain intensity. The secondary outcomes will be abdominal pain frequency, stool consistency, other gastrointestinal symptoms, school performance, and psychological functioning associated with FAPD, parent’s work absenteeism associated with FAPD of a child, health-related quality of life, compliance, growth, and adverse events. The findings will be published in a peer-reviewed journal and submitted to relevant conferences.

**Conclusion:**

This systematic review of rigorous methodological design will update current evidence on the efficacy and safety of using a low-FODMAP diet. However, it may be limited by the quality of the included studies.

## Abstract 2

Supplemental Digital Content is available in the text.

**What is Known**

- A typical low-FODMAP diet is one of the well-documented and recommended treatments in the management of adults with irritable bowel syndrome.
- Evidence for use of a low-FODMAP diet in the management of functional abdominal pain disorders (FAPD) in children is limited.

**What is New**

- This systematic review of rigorous methodological design will update current evidence on the efficacy and safety of using a low-FODMAP diet in children.
- This review will assess the quality of recent randomized controlled trials performed in children with FAPD.

**Translational Impact**

The results of this systematic review may guide future clinical practice guideline development and treatment in regard to dietary management of children with FAPD.

The worldwide pooled global prevalence of functional abdominal pain disorders (FAPD) in children aged 4–18 years was 13.5% in 2015, among which irritable bowel syndrome (IBS) was the most common disorder (8.8%) (1). The prevalence across studies varied from 1.6% to 41.2%. The available pharmacological and nutritional interventions for the management of FAPD in children are limited. A diet low in fermentable oligosaccharides, disaccharides monosaccharides, and polyols (FODMAPs) is widely used in adults and children with functional abdominal pain, despite limited available evidence (2). The rationale for use of a low-FODMAP diet is based on the assumption that a decrease in the short-chain fermentable carbohydrate load both prevents the osmotic effect of FODMAPs, resulting in a decrease in small intestine water volume, and limits the exaggerated fermentation of FODMAPs by colonic microbiota and associated gas production, which may alleviate recurrent abdominal pain (3). A typical low-FODMAP diet should consist of 3 stages: an elimination of most FODMAPs (oligosaccharides [fructans and galactooligosaccharides], polyols [sorbitol or mannitol], excess fructose, and lactose) to the established cutoff levels (4), a rechallenge to identify FODMAP triggers of symptoms with thresholds, and the development of an individual modified low-FODMAP diet based only on identified FODMAP triggers (5). However, in the pediatric population, a simplified approach (i.e., elimination of only some food groups with high FODMAP content or a few targeted FODMAPs—a bottom up approach) is commonly used to avoid over-restriction (5, 6). For a summary of definitions of low-FODMAP diets reported in various publications in the literature, see Table 1.

**TABLE 1.** Summary of the Definitions of a Low-FODMAP Diet

Author, year	Term	The authors’ description
Shepherd and Gibson, 2006 (7)	**A fructose malabsorption diet**	A dietary strategy with the avoidance of foods with high free fructose (>0.5 g/100 g fructose in excess of glucose) and short-chain fructans (>0.5 g per serving) content, limitation of the total fructose load (with or without glucose), encouragement of foods in which glucose was balanced with fructose, and coingestion of free glucose with high “free fructose” foods. Sometimes described as an initial (“primitive”) version of a low-FODMAP diet.
Croagh et al, 2007 (8)	**FODMAP (fermentable oligo-, di-, and mono-saccharides and polyols**)	A group of poorly absorbed short-chain carbohydrates and sugar alcohols (polyols), that include fructose and lactose (if fructose and lactose malabsorbtion is/are present), fructans (oligosaccharides of fructose), and polyols (such as sorbitol) (8)
Shepherd et al, 2008 (9)	**A low FODMAP diet**	A diet which restricts quantities of all FODMAP and in particular fructans, fructose, and foods in which free fructose greatly exceeds free glucose. In a study: diet with exclusion of fruits containing fructose in excess of glucose, fructan-containing vegetables, wheat-based products, sorbitol-containing products, raffinose-containing foods, and lactose containing foods (in case of lactose malabsorption proven with breath hydrogen testing following a load of 50 g).
Tuck et al, 2014 (10) and Whelan et al, 2018 (11)	**“Individual modified low-FODMAP diet”** (10) or **“modified-FODMAP diet”** (11)	A stage of low-FODMAP diet in which patient reintroduces any foods well tolerated and only limit those foods resulting in symptoms (10); restriction is continued [keeping the type and amount of FODMAP intake below the tolerance threshold], but those FODMAP/foods that did not induce symptoms during the second stage are now included (11).
Halmos, 2017 (5)	**Bottom-up approach**	An approach to implement a low-FODMAP diet with a reduction of foods with very large amount or specific FODMAP, then continued restriction to tolerance level (4–8 weeks).
	**Top-down approach**	An approach to implement a low-FODMAP diet with an over restriction of all or most FODMAP to the cutoff levels set by Monash University, then liberalization of diet to tolerance level (4–8 weeks).
Whelan et al, 2018 (11), Halmos and Gibson, 2019 (12), and Mitchell et al, 2019 (13)	**A 3-step low-FODMAP diet**	A low-FODMAP diet that consist of: (1) FODMAP restriction (for 4–8 weeks (11) [or 2–6 weeks (13)] FODMAP intake is reduced to below the tolerance threshold), (2) FODMAP reintroduction (for 6–10 weeks (11) [or 6–8 weeks (13)], during which a food high in one FODMAP is tested over a 3-day period at increasing doses [with each 3-day period separated by at least 1 day depending upon symptom provocation], an individual challenge with a food high in only 1 FODMAP is used to identify individual tolerance to that whole group of FODMAP [only for fructans several foods are used]) (11), (3) FODMAP personalization [involves development a “modified-FODMAP diet” (11); foods which did not induce symptoms are reintroduced back, food which induce mild symptoms may be reintroduced in smaller doses or less frequently, and food which are identified to trigger severe symptoms should be avoided (13)]. Categories of FODMAP include oligosaccharides (fructans [oligofructose, inulin, fructo-oligosaccharides] and galacto-oligosaccharides [raffinose, stachyose]), dissacharide (lactose), monosaccharide (fructose [in high concentrations or in excess of glucose]), and polyols (sorbitol, mannitol and lacitol, xylitol, erythritol, maltitol).
Monash “The low-FODMAP Diet for Irritable Bowel Syndrome” course (accessed September 2019)	**Strict low-FODMAP diet (2–6 weeks**)	A diet with an exclusion of foods with high or moderate amounts of any of the FODMAP sub-groups (excess fructose, lactose, galactooligosaccharides, fructans, mannitol, and sorbitol); followed by the FODMAP rechallenge (in which patient reintroduce individual FODMAP sub-groups to determine which FODMAP they tolerate and which trigger symptoms) and adapted FODMAP diet (that only restricts foods and FODMAP sub-groups that are poorly tolerated and only to an extent that is absolutely necessary)
	**Simplified low-FODMAP diet**	A low-FODMAP diet with a restriction of only frequently consumed very high FODMAP foods and suspected to be major triggers of their IBS symptoms.
	**An “expanding’ low-FODMAP diet**	A low-FODMAP diet aimed to broaden the patient’s diet to include more low-FODMAP foods, knowing that these should be tolerated
Halmos and Gibson, 2019 (12)	**“New” FODMAP concept**	As the often-quoted list of FODMAP groups (…) was never meant to include all FODMAP but just to cover the vast majority of dietary FODMAP of relevance to dietary manipulation, “New” FODMAP might include additionally: passively absorbed monosaccharides (i.e., xylose), other nondigestible oligosaccharides (i.e., xylo-oligosaccharides), lactulose, nutraceuticals and functional foods (i.e., arabinose), inulin, deficiency of other brush border hydrolases in the small intestine.

FODMAP, fermentable oligosaccharides, disaccharides, monosaccharides, and polyols.

Five previous systematic reviews (in children, or children and adults) (2,14–17) found limited evidence with regard to use of a low-FODMAP diet (1 randomized controlled trial [1 RCT]) (18) and a fructose-restricted diet [1 RCT] (19) for the management of FAPD in children. In children with recurrent abdominal pain (16), there was also found to be no effect with use of a lactose-free diet (2 RCTs) (20,21). However, a few more studies have since been published in the last 2 years.

We aim to systematically update evidence on the efficacy and safety of using a low-FODMAP diet (preferably a 3-step low-FODMAP diet but also only a strict low-FODMAP diet or restriction of individual FODMAPs) for the management of children with FAPD. Each low-FODMAP diet or individual FODMAP restriction will be assessed separately.

## METHODS

This protocol follows the Preferred Reporting Items for Systematic review and Meta-Analysis Protocols (PRISMA-P) guidelines for systematic review protocols (for checklist, see Supplemental Digital Content 1, http://links.lww.com/PG9/A28). The *Cochrane Handbook for Systematic Review of Intervention Version 6.1* (22) and PRISMA guidelines for undertaking and reporting the results of a systematic review and meta-analysis (23) will be followed at each stage of this review.

### Eligibility Criteria

#### Type of Population

Studies involving children of any age diagnosed with any of the FAPD, according to established Rome Criteria (I-IV) (24–27) or following the Apley Criteria (28) or other definition provided by the authors of the original study, will be included. If feasible, each FAPD will be assessed separately, as the signs and symptoms among them differ (29). Trials performed in children with comorbidities, as well as studies performed in adults, will be excluded.

#### Type of Intervention and Comparator

Studies using a low-FODMAP diet compared with any type of comparator (i.e., standardized [i.e., average national] or other diet or no intervention) will be eligible for inclusion. Trials that assessed the restriction of individual FODMAPs (i.e., lactose-free diet) also will be included, as in the pediatric population, such or similar approaches (i.e., elimination of only some food groups) are commonly used to avoid over-restriction (5,6). Each type of a low-FODMAP diet will be assessed separately to avoid bias associated with the mixing of dissimilar interventions (30). Studies assessing the effects of a gluten-free diet will not be considered for this review, as this intervention does not exclude any of the FODMAPs.

#### Type of Studies

Only RCTs, including those of crossover design (regardless of language, study duration, settings, and length of follow-up), will be included. As evidence for the employed population, intervention, comparison, outcomes, and studies (PICOS)’ question is generally scarce (31), and to avoid bias associated with any exclusion of unpublished results, abstracts and letters to the editor, preliminary analyses will be included if available data are sufficient for the analyses. Ongoing studies will be excluded; however, they will be reported in the “Characteristics of ongoing studies” table.

#### Type of Outcomes Measures

The primary outcome will be the difference in abdominal pain intensity between the intervention and control groups, measured ideally with any validated measurement instrument (e.g., Visual Analogue Scale). However, if lacking, any method reported by the authors will be accepted.

Secondary outcomes will include change in pain frequency; stool consistency; other gastrointestinal symptoms (assessed as single symptoms or as group of gastrointestinal symptoms); school performance (i.e., school attendance) associated with FAPD symptoms; psychological functioning (i.e., anxiety) associated with FAPD symptoms; parent’s work absenteeism associated with FAPD symptoms in child; health-related quality of life; adherence to the diet (compliance); growth (subjects’ body weight and length); adverse events (measured as a proportion of the participants reporting an adverse event throughout the trial, extracted with characteristics). If feasible, the reported measurement instruments will be extracted.

### Information Sources

The Cochrane Central Register of Controlled Trials (CENTRAL, the Cochrane Library), MEDLINE (PubMed), and EMBASE databases will be searched for relevant studies from inception to the date of completion of this review. A weekly e-mail update of new search results in PubMed will be set up. The search terms for this review will be based on the 3 previous similar systematic reviews (32–34). The search strategy will include use of the Cochrane Collaboration’s validated filters (http://work.cochrane.org/rct-filters-different-databases) for identifying RCTs in PubMed and EMBASE, which will be combined with the main Medical Subject Headings (MeSH) and text key-words (see Supplemental Digital Content 2, http://links.lww.com/PG9/A27). The search will be carried out independently by 2 reviewers.

#### Other Sources

The ClinicalTrials.gov, National Institute for Health Research (http://www.ukctg.nihr.ac.uk/default.aspx), and Turning Research into Practice (https://www.tripdatabase.com/) websites also will be screened to identify unpublished and ongoing trials. Moreover, the International Clinical Trial Registry Platform (http://apps.who.int/trialsearch/), which is a more comprehensive trial registry, will be considered, if available (at the time of the writing of this protocol, it is temporarily unavailable).

### Study Records

#### Study Selection Process

Two reviewers (AH, AS) will independently screen the title, abstract, and keywords of every study identified with the search strategy and other source records using the EndNote X9 Computer program [Version 9.3.3. Philadelphia, The Clarivate Analytics, 2020]. For eligible and unclear studies, relevant systematic reviews and reviews, the full texts will be retrieved and assessed against eligibility criteria (not for conference abstracts).

#### Data Collection and Management Process

Data extraction will be performed using data-extraction forms developed by the reviewers. Relevant data will be extracted by one reviewer (AS) and independently checked by the second reviewer (AH). Reviewers will attempt to contact the authors of the original study whenever reported data are insufficient for planned analysis. Any discrepancies with regard to the study selection and extraction will be resolved through discussion, and, if necessary, another review author (HS) will be consulted. Excluded RCTs with reasons for exclusion will be reported in a “Characteristics of excluded studies” table. The process of selection will be presented as a PRISMA flow diagram.

### Data Items

Data will be extracted by one reviewer (AS) and independently checked by the second reviewer (AH). Any discrepancies will be resolved by discussion until a consensus is reached.

The reviewers will independently extract the following data from the included trials:

- Authors, country, and year of publication;
- Methods (study design).
- Participants (number, age, sex, applied diagnostic criteria [if available], IBS subtype [if feasible], and other population characteristics associated with this analysis).
- Intervention (description of intervention; duration of intervention; additional characteristics, if needed; number of participants in the intervention group).
- Description of the comparator and number of participants in the control group.
- Methods of introduction of the intervention and comparator.
- Duration of follow-up.
- Registered protocol and pre-specified outcomes.
- Outcomes (data for primary and secondary outcomes, i.e., units of measurement, time points). For dichotomous outcomes, if feasible, the total number of participants and the number of participants who experienced the event will be extracted. For continuous outcomes, if feasible, the total number of participants and the mean differences and standard deviations will be extracted. Outcomes data, if feasible will be extracted using the Review Manager [Computer program. Version 5.2 Copenhagen, The Cochrane Collaboration, 2012].
- Sample size.
- Conflict of interests.

Data items extracted from the included trials will be reported in a “Characteristics of included studies” table.

### Risk of Bias in Individual Studies

The risk of bias of all included trials will be assessed independently by 2 reviewers (AS, AH) using the Cochrane Collaboration’s tool for assessing risk of bias (including: adequacy of sequence generation, allocation concealment, and blinding of participants, personnel and outcome assessors; incomplete outcome data are addressed, free of selective outcome reporting, and free of other sources of bias). The risk will be classified as “high,” “low,” or “unclear,” following the *Cochrane Handbook for Systematic Review of Intervention Version 5.1* (35). Discrepancies between the reviewers will be resolved by discussion until a consensus is reached; in case of disagreement, the other reviewer (HS) will be consulted.

### Data Synthesis

If feasible for analysis, data will be entered by one reviewer (AS) into Review Manager [Computer program. Version 5.2 Copenhagen, The Cochrane Collaboration, 2012] and checked for accuracy by the other reviewer (AH). Relative risk (RR) between the experimental and control groups with a 95% confidence interval (CI) will be used for dichotomous outcomes, and mean difference (MD) with a 95% CI will be used for continuous outcomes. All analyses will be based on the random-effects model. In the event of substantial unexplained heterogeneity of the analyzed population, intervention, comparators, and outcomes, the results of the trials will not be pooled in a meta-analysis but rather assessed and described narratively (30).

### Heterogeneity

If feasible, heterogeneity will be quantified by χ^2^ and I^2^, which can be interpreted as the percentage of the total variation between studies that is attributable to heterogeneity rather than to chance. A value of 0% to 40% indicates heterogeneity that may not be important, and larger values show increasing heterogeneity.

### Subgroup Analysis

Whenever feasible, for the primary outcome, we will carry out subgroup analyses based on factors that potentially may influence it, such as age of participants; sex of participants; applied diagnostic criteria; duration of intervention; comparators; concomitant medications; and, in the case of the IBS population, IBS subtypes.

### Meta-bias

The publication bias will be assessed by the generation of funnel plots as proposed by Egger et al (36), if at least 10 studies are included.

### Confidence in Cumulative Evidence

If feasible, the quality of the evidence according to Grading of Recommendations Assessment, Development and Evaluation (GRADE) methodology (37) will be performed, using the GRADEPro Guideline Development Tool.

### Dissemination

The findings of this review will be published in a peer-reviewed journal in accordance with PRISMA (23). Abstracts will be submitted to relevant national and international conferences.

## CONCLUSION

This systematic review will update current RCT evidence on the efficacy and safety of using a low-FODMAP diet in the management of childhood FAPD. However, it may be limited by the number and quality of the included studies. It is the hope that this systematic review will guide future clinical practice guideline development and treatment in regard to dietary management of children with FAPD.

## Supplementary Material



## References

[1] Korterink JJ Diederen K Benninga MA. Epidemiology of pediatric functional abdominal pain disorders: a meta-analysis. PLoS One. 2015; 10:e0126982 25992621 10.1371/journal.pone.0126982 PMC4439136

[2] Newlove-Delgado TV Martin AE Abbott RA. Dietary interventions for recurrent abdominal pain in childhood. Cochrane Database Syst Rev. 2017; 3:CD010972 28334433 10.1002/14651858.CD010972.pub2 PMC6464236

[3] Varjú P Farkas N Hegyi P. Low fermentable oligosaccharides, disaccharides, monosaccharides and polyols (FODMAP) diet improves symptoms in adults suffering from irritable bowel syndrome (IBS) compared to standard IBS diet: a meta-analysis of clinical studies. PLoS One. 2017; 12:e0182942 28806407 10.1371/journal.pone.0182942 PMC5555627

[4] Varney J Barrett J Scarlata K. FODMAPs: food composition, defining cutoff values and international application. J Gastroenterol Hepatol. 2017; 32(suppl 1):53–61 10.1111/jgh.13698 28244665

[5] Halmos EP. When the low FODMAP diet does not work. J Gastroenterol Hepatol. 2017; 32(suppl 1):69–72 28244666 10.1111/jgh.13701

[6] Iacovou M. Adapting the low FODMAP diet to special populations: infants and children. J Gastroenterol Hepatol. 2017; 32(suppl 1):43–45 28244674 10.1111/jgh.13696

[7] Shepherd SJ Gibson PR. Fructose malabsorption and symptoms of irritable bowel syndrome: guidelines for effective dietary management. J Am Dietetic Assoc. 2006; 106:1631–1639 10.1016/j.jada.2006.07.010 17000196

[8] Croagh C Shepherd SJ Berryman M Muir JG Gibson PR. Pilot study on the effect of reducing dietary FODMAP intake on bowel function in patients without a colon. Inflamm Bowel Dis. 2007; 13:1522–1528 17828776 10.1002/ibd.20249

[9] Shepherd S Parker F Muir J Gibson P. Dietary triggers of abdominal symptoms in patients with irritable bowel syndrome: randomized placebo-controlled evidence. Clinic Gastroenterol Hepatol. 2008; 6:765–771 10.1016/j.cgh.2008.02.058 18456565

[10] Tuck CJ Muir JG Barrett JS Gibson PR. Fermentable oligosaccharides, disaccharides, monosaccharides and polyols: role in irritable bowel syndrome. Expert Rev Gastroenterol Hepatol. 2014; 8:819–834 24830318 10.1586/17474124.2014.917956

[11] Whelan K Martin LD Staudacher HM Lomer MCE. The low FODMAP diet in the management of irritable bowel syndrome: an evidence-based review of FODMAP restriction, reintroduction and personalisation in clinical practice. J Hum Nutr Diet. 2018; 31:239–255 29336079 10.1111/jhn.12530

[12] Halmos EP Gibson PR. Controversies and reality of the FODMAP diet for patients with irritable bowel syndrome. J Gastroenterol Hepatol. 2019; 34:1134–1142 30945376 10.1111/jgh.14650

[13] Mitchell H Porter J Gibson PR Barrett J Garg M. Review article: implementation of a diet low in FODMAPs for patients with irritable bowel syndrome-directions for future research. Aliment *P*harmacol *T*herapeut. 2019; 49:124–139 10.1111/apt.15079 30589971

[14] Abbott RA Martin AE Newlove-Delgado TV. Recurrent abdominal pain in children: summary evidence from 3 systematic reviews of treatment effectiveness. J Pediatr Gastroenterol Nutr. 2018; 67:23–33 29470291 10.1097/MPG.0000000000001922

[15] Marsh A Eslick EM Eslick GD. Does a diet low in FODMAPs reduce symptoms associated with functional gastrointestinal disorders? A comprehensive systematic review and meta-analysis. Eur J Nutr. 2016; 55:897–906 25982757 10.1007/s00394-015-0922-1

[16] Rutten JM Korterink JJ Venmans LM. Nonpharmacologic treatment of functional abdominal pain disorders: a systematic review. Pediatrics. 2015; 135:522–535 25667239 10.1542/peds.2014-2123

[17] Turco R Salvatore S Miele E. Does a low FODMAPs diet reduce symptoms of functional abdominal pain disorders? A systematic review in adult and paediatric population, on behalf of Italian Society of Pediatrics. Ital J Pediatr. 2018; 44:53 29764491 10.1186/s13052-018-0495-8 PMC5952847

[18] Chumpitazi BP Cope JL Hollister EB. Randomised clinical trial: gut microbiome biomarkers are associated with clinical response to a low FODMAP diet in children with the irritable bowel syndrome. Journal article; Randomized Controlled Trial; Research support, N.I.H., Extramural; Research support, non-U.S. Gov’t. Aliment Pharmacol Ther. 2015; 42:418–427 26104013 10.1111/apt.13286 PMC4514898

[19] Wirth S Klodt C Wintermeyer P. Positive or negative fructose breath test results do not predict response to fructose restricted diet in children with recurrent abdominal pain: results from a prospective randomized trial. Klin Padiatr. 2014; 226:268–273 25153911 10.1055/s-0034-1383653

[20] Dearlove J Dearlove B Pearl K. Dietary lactose and the child with abdominal pain. Br Med J (Clin Res Ed). 1983; 286:1936 10.1136/bmj.286.6382.1936 PMC1548276 6407644

[21] Lebenthal E Rossi TM Nord KS. Recurrent abdominal pain and lactose absorption in children. Pediatrics. 1981; 67:828–832 7195004

[22] Higgins JPT Thomas J Chandler J. Cochrane Handbook for Systematic Reviews of Interventions version 6.2. (updated February 2021). Cochrane; 2021. Available at: www.training.cochrane.org/handbook

[23] Liberati A Altman DG Tetzlaff J. The PRISMA statement for reporting systematic reviews and meta-analyses of studies that evaluate health care interventions: explanation and elaboration. Ann Intern Med. 2009; 151:W65–W94 19622512 10.7326/0003-4819-151-4-200908180-00136

[24] Hyams JS Di Lorenzo C Saps MS. Childhood functional gastrointestinal disorders: child/adolescent. Gastroenterology. 2016; 150:1456–1468.e2

[25] Drossman DA. The Functional Gastrointestinal Disorders: Diagnosis, Pathophysiology, and Treatment: A Multinational Consenus. 1994, Little brown

[26] Rasquin A Di Lorenzo C Forbes D. Childhood functional gastrointestinal disorders: child/adolescent. Gastroenterology. 2006; 130:1527–1537 16678566 10.1053/j.gastro.2005.08.063 PMC7104693

[27] Rasquin-Weber A Hyman PE Cucchiara S. Childhood functional gastrointestinal disorders. Gut. 1999; 45(suppl 2):Ii60–Ii68 10457047 10.1136/gut.45.2008.ii60 PMC1766693

[28] Apley J Naish N. Recurrent abdominal pains: a field survey of 1,000 school children. Arch Dis Child. 1958; 33:165–170 13534750 10.1136/adc.33.168.165 PMC2012205

[29] Saps M van Tilburg MA Lavigne JV. Recommendations for pharmacological clinical trials in children with irritable bowel syndrome: the Rome foundation pediatric subcommittee on clinical trials. Neurogastroenterol Motil. 2016; 28:1619–1631 27477090 10.1111/nmo.12896

[30] McKenzie JE Brennan SE Ryan RE. Higgins JPT Thomas J Chandler J. Chapter 9: Summarizing study characteristics and preparing for synthesis. Cochrane Handbook for Systematic Reviews of Interventions version 6.0. 2019, Cochrane. (updated July 2019)

[31] Scherer RW Saldanha IJ. How should systematic reviewers handle conference abstracts? A view from the trenches. Syst Rev. 2019; 8:264 31699124 10.1186/s13643-019-1188-0 PMC6836535

[32] Pensabene L Salvatore S Turco R. Low FODMAPs diet for functional abdominal pain disorders in children: critical review of current knowledge. J Pediatr (Rio J). 2019; 95:642–656 31028745 10.1016/j.jped.2019.03.004

[33] Schumann D Klose P Lauche R. Low fermentable, oligo-, di-, mono-saccharides and polyol diet in the treatment of irritable bowel syndrome: a systematic review and meta-analysis. Nutrition. 2018; 45:24–31 29129233 10.1016/j.nut.2017.07.004

[34] Zeevenhooven J Timp ML Singendonk MMJ. Definitions of pediatric functional abdominal pain disorders and outcome measures: a systematic review. J Pediatr. 2019; 212:52–59.e16 31277898 10.1016/j.jpeds.2019.04.048

[35] Higgins JPT Green S. Cochrane Handbook for Systematic Reviews of Interventions Version 5.1.0. [updated March 2011]. The Cochrane Collaboration; 2011. Available at: www.handbook.cochrane.org

[36] Egger M Davey Smith G Schneider M. Bias in meta-analysis detected by a simple, graphical test. BMJ. 1997; 315:629–634 9310563 10.1136/bmj.315.7109.629 PMC2127453

[37] Guyatt GH Oxman AD Vist GE. GRADE Working Group GRADE: an emerging consensus on rating quality of evidence and strength of recommendations. BMJ. 2008; 336:924–926 18436948 10.1136/bmj.39489.470347.AD PMC2335261

[38] Gibson PR Shepherd SJ. Evidence-based dietary management of functional gastrointestinal symptoms: The FODMAP approach. J Gastroenterol Hepatol. 2010; 25:252–258 20136989 10.1111/j.1440-1746.2009.06149.x

