# Supplementary material for: A Low-FODMAP Diet in the Management of Children With Functional Abdominal Pain Disorders: A Protocol of a Systematic Review
Source: JPGN Rep. 2021 Mar 30;2(2):e065. doi: 10.1097/PG9.0000000000000065 (PMC10191571; doi:10.1097/PG9.0000000000000065)
Supplement: Supplementary file 1 [file pg9-2-e065-s001.pdf]

Supplemental Digital Content. Search strategy.

a) MEDLINE via PubMed

((((FODMAP[tiab] OR FODMAPS[tiab] OR (fermentable oligosaccharides, disaccharides, monosaccharides and polyols[tiab])) OR ((fermentable, poorly absorbed, short-chain carbohydrates[tiab]) OR (fermentable oligosaccharides, disaccharides, monosaccharides and polyols[tiab])) OR ((lactose-free diet[tiab]) OR (fructose[tiab]) OR (fructans[tiab]) OR (sorbitol[tiab]))))) AND (((("Colonic Diseases, Functional"[Mesh] OR "Irritable Bowel Syndrome"[Mesh] OR irritable bowel[tiab] OR IBS[tiab] OR abdominal discomfort[tiab] OR abdominal migraine[tiab] OR functional dyspepsia[tiab])) OR (("Abdomen"[Majr:noexp] AND "Pain"[Majr:noexp])) OR (("Abdominal Pain"[Mesh] OR "Gastrointestinal Diseases"[Mesh] OR gastrointestinal diseas*[tiab] OR gastrointestinal disorder*[tiab] OR abdominal pain*[tiab]) AND (functional[tiab] OR continuous[tiab] OR continue[tiab] OR recurrent[tiab] OR chronic[tiab] OR episodic[tiab])))) AND ((((randomized controlled trial[pt] OR controlled clinical trial[pt] OR randomized[tiab] OR placebo[tiab] OR clinical trials as topic[mesh:noexp] OR randomly[tiab] OR trial[ti] NOT (animals[mh] NOT humans[mh])))))

b) EMBASE

('low fodmap diet'/exp OR 'low fodmap diet' OR 'lactose free diet':de
OR 'fructan':de OR 'fructose':de OR 'sorbitol':de) AND ('irritable colon'/exp
OR 'irritable colon' OR 'colon disease':de OR 'intestine function disorder':de
OR 'abdominal discomfort':de OR 'abdominal migraine':de OR 'dyspepsia':de
OR 'abdominal pain':de OR 'digestive system function disorder':de OR 'recurrent abdominal pain':de) AND ('crossover procedure':de OR 'double-blind procedure':de
OR 'randomized controlled trial':de OR 'single-blind procedure':de
OR random*:de,ab,ti OR factorial*:de,ab,ti OR crossover*:de,ab,ti OR
((cross NEXT/1 over*):de,ab,ti) OR placebo*:de,ab,ti OR
((doubl* NEAR/1 blind*):de,ab,ti) OR ((singl* NEAR/1 blind*):de,ab,ti)
OR assign*:de,ab,ti OR allocat*:de,ab,ti OR volunteer*:de,ab,ti)

c) COCHRANE

ID	SearchHits
#1	MeSH descriptor: [Colonic Diseases, Functional] explode all trees
#2	MeSH descriptor: [Irritable Bowel Syndrome] explode all trees
#3	irritable bowel or IBS:ti,ab,kw
#4	abdominal near/3 discomfort:ti,ab,kw
#5	abdominal near/3 migraine:ti,ab,kw
#6	MeSH descriptor: [Abdominal Pain] explode all trees
#7	MeSH descriptor: [Gastrointestinal Diseases] explode all trees
#8	#6 or #7

#9 functional or continuous or continue or recurrent or chronic or episodic:ti,ab,
#10 #8 and #9
#11 (abdominal pain* or gastrointestinal diseas* or gastrointestinal disorder* or dyspepsia) near/3 (functional or continuous or continue or recurrent or chronic or episodic):ti,ab,kw
#12 #1 or #2 or #3 or #4 or #5 or #10 or #11
#13 fermentable oligosaccharides, disaccharides, monosaccharides and polyols:ti,ab
#14 FODMAP:ti,ab
#15 low FODMAP diet:ti,ab
#16 fermentable, poorly absorbed, short-chain carbohydrates:ti,ab
#17 lactose-free diet:ti,ab
#18 fructose:ti,ab
#19 fructans:ti,ab
#20 sorbitol:ti,ab
#21 #13 or #14 or #15 or #16 or #17 or #18 or #19 or #20
#22 #12 and #21
